# First report of *Longidorus mindanaoensis* Coomans, De Ley, Jimenez and De Ley, 2012 (Nematoda: Longidoridae) From a Mangrove Forest in Vietnam

**DOI:** 10.21307/jofnem-2019-064

**Published:** 2019-09-17

**Authors:** Thi Duyen Nguyen, Huu Tien Nguyen, Thi Mai Linh Le, Neriza Nobleza, Quang Phap Trinh

**Affiliations:** 1 Institute of Ecology and Biological Resources, Vietnam Academy of Sciences and Technology, 18 Hoang Quoc Viet, Cau Giay, 100000 Hanoi, Vietnam; 2 Graduate University of Science and Technology, Vietnam Academy of Sciences and Technology, 18 Hoang Quoc Viet, Cau Giay, 100000 Hanoi, Vietnam; 3 College of Agriculture, Mindanao State University, Main Campus, Marawi City, 9700 Lanao del Sur, The Philippines

**Keywords:** Ca Mau, Dat Mui, deepwater, marine nematode, needle nematode, Ngoc Hien, plant-parasitic nematode, saline sea sediment

## Abstract

*Longidorus mindanaoensis* was recovered from a mangrove forest in Vietnam. The recovered population is in general morphological agreement with the type population, and the characters of pharyngeal bulb, i.e. the same unique pattern of pharyngeal glands nuclei as well as the lip region morphology, amphidial fovea shape and size and position of vulva corroborated its identity. Molecular studies of the recovered population using D2–D3 expansion segments of large subunit ribosomal DNA (LSU rDNA D2–D3) revealed the D2–D3 sequence of recovered population is 99.6% similar to the sequence of the type population. A new morphometric range for body size was recorded for the species based upon present Vietnamese population, and present study emphasized the diversity of *Longidorus* spp. in Vietnam could be higher than previously assumed.

*Longidorus* spp. (needle nematodes) are migratory ectoparasitic nematodes. Besides their direct damages by direct feeding from root cells, some species could also transmit plant pathogenic viruses (Taylor and Brown, 1997). Currently, only one valid species (*Longidorus elongatus* (de Man, 1876) Micoletzky, 1927) has been reported in association with peanut (*Arachis hypogaea* L.) from Vietnam.

During this study, a population of the genus *Longidorus* was recovered from saline sea sediments in a mangrove forest in Vietnam (GPS coordinates N: 8°38′09.902′; E: 104°44′31.178′). Nematodes were extracted from soil samples using the tray method (Whitehead and Hemming, 1965). The specimens were killed, fixed in TAF, and transferred to glycerin according to Seinhorst (1959). The measurements and preparing the microphotographs were performed using a Carl Zeiss Axio Lab.A1 light microscope equipped with a ZEISS Axiocam ERc5s digital camera (Nguyen et al., 2017). For molecular phylogenetic analysis, the D2–D3 expansion segments of LSU rDNA were amplified using the primers D2A and D3B (5′-ACAAGTACCGTGGGGAAAGTTG-3′ and 5′-TCGGAAGGAACCAGCTACTA-3′) (De Ley et al., 1999). The newly obtained sequence was compared with previously submitted sequences into the GenBank database (Altschul et al., 1997) using BLAST search. Multiple alignments were made using MUSCLE and Modeltest was used to select the best fit model in MEGA 6 (Tamura et al., 2013). MrBayes 3.2.6 (Huelsenbeck and Ronquist, 2001) in Geneious R11 (www.geneious.com) was used to infer the Bayesian phylogenetic tree with 10^6^ generations of Markov chains (4 runs, 20% burn-in) (Nguyen et al., 2019).

The morphological comparisons revealed the studied population belongs to *Longidorus mindanaoensis* (Coomans et al., 2012) that was recovered from the same habitat in the Philippines by Coomans et al. (2012). The morphological characters and measurements of the Vietnamese population of *L. mindanaoensis* (Fig. 1, Table 1) are in agreement with the original description of the species by Coomans et al. (2012). Some unique morphological and morphometric characters like the shape of lip region and amphidial fovea, the characters of the esophageal bulb (its small size and shape as well as the arrangement of glands nuclei) and the position of vulva delimiting the species well corroborated the identity of the species. However, new morphometric data ranges were recorded for the species as follows: the females and males of the presently studied population of *L. mindanaoensis* from Vietnam are smaller than the type population (Table 1), and therefore, related indices, including a, b are also relatively smaller compared with the data given for the type population. The present observation is in accordance with the results of Coomans et al. (2012) and Archidona-Yuste et al. (2016) showing the body length of *Longidorus* spp. could be highly variable (Coomans et al., 2012; Archidona-Yuste et al., 2016).

**Table 1.** Measurements of *Longidorus mindanaoensis* (Coomans et al., 2012) from Vietnam and the Philippines.

	Longidorus mindanaoensis (Vietnamese population)		Longidorus mindanaoensis (Coomans et al., 2012)	
	Female	Male	Female	Male
*n*	8	5	32	22
*L*	4.82 ± 0.45 (3.95–5.26)	4.44 ± 0.18 (4.27–4.63)	6.59 ± 0.48 (5.44–7.37)	6.14 ± 0.23 (5.60–6.45)
*a*	59 ± 10.5 (46–70)	61 ± 14.2 (48–85)	87 ± 5.2 (75–97)	93 ± 4.3 (84–102)
*b*	10.7 ± 0.9 (9.3–11.8)	10.5 ± 0.5 (10.0–11.2)	13.2 ± 1.15 (11–16.8)	12.8 ± 0.91 (11–14.7)
*c*	204 ± 40 (143–250)	182 ± 4.3 (177–188)	241 ± 31 (176–296)	197 ± 19 (167–234)
*c'*	0.5 ± 0.1 (0.4–0.6)	0.6 ± 0.0 (0.5–0.6)	0.58 ± 0.04 (0.49–0.67)	0.68 ± 0.05 (0.58–0.79)
*V*	36 ± 1.8 (32–38)	–	36 ± 1.6 (33–39)	–
Odontostyle	126 ± 3.5 (122–133)	126 ± 5.8 (116–131)	134 ± 5.4 (117–142)	134 ± 5.9 (120–145)
Odontophore	90 ± 10.4 (75–104)	89 ± 8.1 (76–97)	70 ± 5.7 (61–80)	68 ± 7.3 (48–83)
Anterior end to nerve ring	217 ± 15.0 (192–236)	205 ± 10.1 (197–222)	217 ± 9 (195–233)	216 ± 8 (195–240)
Pharynx	453 ± 30 (419–511)	424 ± 30 (380–455)	493 ± 35 (400–584)	480 ± 32 (413–540)
Max. body diam. (MBD)	84 ± 12.5 (70–106)	75 ± 13.9 (54–92)	76 ± 5.6 (66–86)	66 ± 3.1 (60–72)
Body diam. at anus/cloaca (ABD)	50 ± 5.6 (44–58)	42 ± 1.6 (40–44)	48 ± 2.2 (44–52)	46 ± 2 (42–50)
Tail length	24 ± 5.0 (21–36)	24 ± 1.0 (23–26)	28 ± 2.5 (22–33)	31 ± 3.1 (27–38)
Anterior genital tract	862 ± 139 (656–1,045)	–	1,009 ± 174 (653–1,259)	–
Posterior genital tract	964 ± 140 (754–1,121)	–	1,053 ± 168 (733–1,324)	–
Spicule length	–	90 ± 4.8 (84–97)	–	87 ± 5 (73–97)
Spicule width	–	13.3 ± 1.1 (11.8–14.5)	–	–
Prerectum	284 ± 28 (266–348)	359 ± 30 (309–389)	372 ± 112 (174–740)	532 ± 107.5 (340–714)

Note: All measurements are in μm (except for L in mm) and in the form: mean ± s.d. (range).

**Figure 1:**
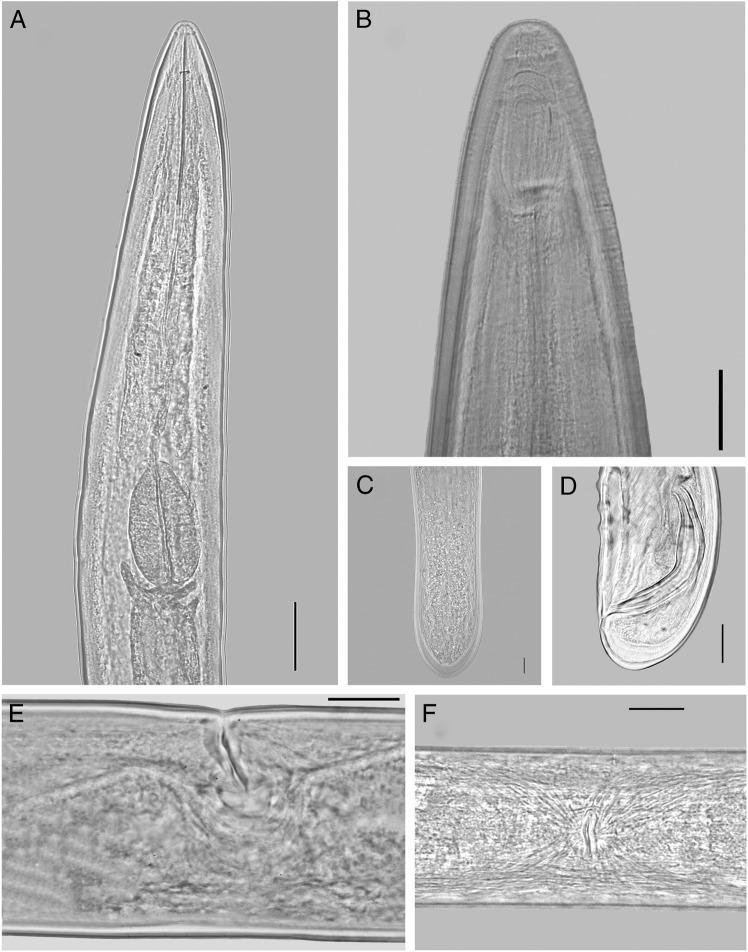
Figure 1: *Longidorus mindanaoensis* (Coomans et al., 2012) from Vietnam. (A–F) Female. (A) Esophageal region; (B) Anterior region; (C) Posterior region; (E) Vulval region; (F) Vulva region, ventral view. (D) Male posterior region. (Scale: (A) 50 µm; (B–F) 20 µm).

The D2–D3 sequence of our population of *L. mindanaoensis* (accession number: MN071244) from Vietnam was 805 bp long. It had a 99.6% identity to the same sequence of the type population and three different nucleotides were detected between both populations. This variation could be explained by intraspecies variation in this genomic region and is recently observed for several other longidorids and trichodorids (Pedram et al., 2017; Fouladvand et al., 2019). It had a 69.3 to 80.7% identity with sequences available in the GenBank database (154-265 different nucleotides). In the inferred Bayesian phylogenetic tree, the newly generated sequence and the original sequence of the type population formed a basal clade to some other selected sequences of the genus (Fig. 2). This genomic fragment has already used in molecular phylogenetic analyses of longidorids and has successfully delimited several species belonging to cryptic complexes (Pedram et al., 2012; Gutiérrez-Gutiérrez et al., 2013; Pedram et al., 2017; Zhao et al., 2017; Fouladvand et al., 2019).

**Figure 2:**
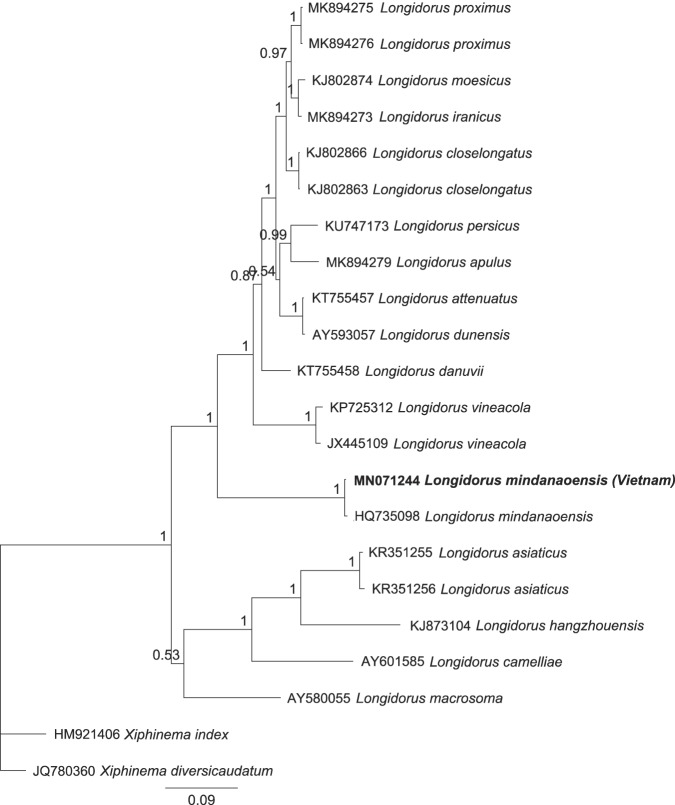
Figure 2: Bayesian phylogenetic tree generated using D2–D3 expansion segments of *Longidorus mindanaoensis* (Coomans et al., 2012) from Vietnam using *Xiphinema index* (Thorne and Allen, 1950) and *Xiphinema diversicaudatum* (Micoletzky, 1927) (Thorne, 1939) as outgroup taxa under the GTR+G evolutionary model.
